# Field surveying data of low-cost networked flood sensors in southeast Texas

**DOI:** 10.1016/j.dib.2023.109504

**Published:** 2023-08-22

**Authors:** Hossein Hariri Asli, Nicholas Brake, Joseph Kruger, Liv Haselbach, Mubarak Adesina

**Affiliations:** a Department of Civil and Environmental Engineering, Lamar University, Beaumont, TX 77710, United States; b Center for Resiliency, Lamar University, Beaumont, TX 77710, United States; c Department of Earth and Space Sciences, Lamar University, Beaumont, TX 77710, United States

**Keywords:** Flood monitoring, Flood warning system, Low-cost networked sensors, Southeast Texas, Flood stage elevation thresholds, Flood resilient community

## Abstract

Floods are common natural disasters worldwide and pose substantial risks to life, property, food production, and natural resources. Effective measures for flood mitigation and warning are essential. Southeast Texas is still at significant risk of flooding, and Lamar University is assisting the region with asset management of a flood sensor network for flooding events. This network provides real-time water stage information. Lamar University developed a survey program to measure elevation and coordinates at each sensor site location to make this data more useful for flood monitoring and mapping. This paper overviews the measurement of the elevation and coordinates of 74 networked flood sensors and various flood stage thresholds at critical points that flood decision-makers can use for reference at each site. In the first phase of this program, these sensors were deployed throughout a 7-county region spanning nearly 6,000 square miles in Southeast Texas. The latitude and longitude of the sensors and their elevations were determined using survey-grade Global Navigation Satellite System (GNSS) technology. Various Continually Operating Reference Stations (CORS) were utilized for post-processing to achieve sub-inch resolution. The flood stage thresholds, water level sensors elevation, and the elevations and positions of other critical surrounding points are viewable to the public through two online repositories and a web-based sensor management dashboard. The data is used to aid with decisions related to road closures or modeling efforts by mitigation decision-makers, emergency managers, and the public, including the Texas Department of Transportation, Houston Transtar, the National Weather Service, and the Sabine River Authority of Texas (SRA).

Specifications Table

**Table:** 

Subject	Engineering.
Specific subject area	Vertical and horizontal positioning of flood networked sensors to monitor and map floods, predict natural hazards, and address the decision-makers' emergency measures.
Type of data	Table Shapefile
How the data were acquired	A Trimble Geo 7X Global Navigation Satellite System (GNSS) handheld device, which employs Trimble H-StarTM technology, and a ZIPLEVEL PRO-2000 High Precision Altimeter were used to determine the coordinates and elevations of the sensors and surrounding critical points. Post-processing of the GNSS data used the Trimble GPS Pathfinder Office 5.9 software. The closest CORS base stations were used for differential corrections and the NAD 1983 (2011) (epoch 2010.00) horizontal datum was used as the geographic coordinate system. Furthermore, orthometric heights were calculated using GEOID 18 which is referenced to the North American Vertical Datum of 1988 (NAVD 88). ArcGIS Pro 3 was used to create a map of the sensors and critical points.
Data format	Raw (.ssf) and post-processed differentially corrected (.cor) files.
Description of data collection	Data were gathered in Southeast Texas watersheds and sub-watersheds in order to monitor and map the elevation and movement of water in the drainages. Vertical and horizontal positions of the 74 flood sensors installed in the first phase of the project and their surrounding critical points, including the node (solar panels, battery, and transmission device), the bottom of the posts that nodes attached (bottom of the node from now on), top of the bank, the bottom of the ditch, the bottom of the bridge's deck, and the center of the road and edges, have been gathered accordingly. Also, the relative elevations between these points are important and were collected.
Data source location	• Institution: Center for Resiliency (CfR), Lamar University • Region: Southeast Texas • County: Jefferson, Orange, Jasper, Newton, Chambers, Hardin, Liberty • Country: United States of America
Data accessibility	Mendeley Data [1]: https://data.mendeley.com/datasets/kwydrvscym Data (Excel): https://data.mendeley.com/public-files/datasets/kwydrvscym/files/285e00cf-4543-4d38-a13b-88866add7abc/file_downloaded Data (CSV): https://data.mendeley.com/public-files/datasets/kwydrvscym/files/8e535476-1f68-4290-9cc8-c64c27e67ae4/file_downloaded https://data.mendeley.com/public-files/datasets/kwydrvscym/files/c97ca779-05b8-4212-a034-fae531521401/file_downloaded Data (Raw data, .zip): https://data.mendeley.com/public-files/datasets/kwydrvscym/files/47c06434-68e2-4c8a-a013-3743d99a9c35/file_downloaded https://data.mendeley.com/public-files/datasets/kwydrvscym/files/dbe2a2fc-6457-4572-b4f5-ab7d9b2a50d2/file_downloaded https://data.mendeley.com/public-files/datasets/kwydrvscym/files/3a90bc31-4c73-47b5-a0cb-cd97746f533d/file_downloaded
	ArcGIS (Spatial features and attribute table, .zip): https://data.mendeley.com/public-files/datasets/kwydrvscym/files/0d0c826f-2015-4295-8b76-9ecacc3639f0/file_downloaded https://data.mendeley.com/public-files/datasets/kwydrvscym/files/cba756da-d9f8-4d05-9f6f-9b1519076d0c/file_downloaded HYDROSHARE [2]: http://www.hydroshare.org/resource/1d1ed97e40024409a866d2164e3e001c Data (Excel): https://www.hydroshare.org/resource/1d1ed97e40024409a866d2164e3e001c/data/contents/Southeast_Texas_Networked_Flood_Monitoring_Sensors.xlsx Data (CSV): https://www.hydroshare.org/resource/1d1ed97e40024409a866d2164e3e001c/data/contents/Southeast_Texas_Networked_Flood_Monitoring_Sensors_CSV2.csv https://www.hydroshare.org/resource/1d1ed97e40024409a866d2164e3e001c/data/contents/Southeast_Texas_Networked_Flood_Monitoring_Sensors_CSV1.csv Data (Raw data, .zip): https://www.hydroshare.org/resource/1d1ed97e40024409a866d2164e3e001c/data/contents/Southeast_Texas_Networked_Flood_Monitoring_Sensors___All_Raw_files___Surveying.zip https://www.hydroshare.org/resource/1d1ed97e40024409a866d2164e3e001c/data/contents/Southeast_Texas_Networked_Flood_Monitoring_Sensors___All_Raw_files___.SSF.zip https://www.hydroshare.org/resource/1d1ed97e40024409a866d2164e3e001c/data/contents/Southeast_Texas_Networked_Flood_Monitoring_Sensors___All_Raw_files___.COR.zip ArcGIS (Spatial features and attribute table, .zip): https://www.hydroshare.org/resource/1d1ed97e40024409a866d2164e3e001c/data/contents/Southeast_Texas_Networked_Flood_Monitoring_Sensors___All_Sites___Shapefile.zip https://www.hydroshare.org/resource/1d1ed97e40024409a866d2164e3e001c/data/contents/Southeast_Texas_Networked_Flood_Monitoring_Sensors___Each_Site_Details___Shapefiles.zip SE TEXAS R.A.I.N [3]: https://setexasrain.onerain.com/

## 1 Value of the Data

• The data is particularly advantageous for emergency managers as it helps them establish a flood alert network in a specific region. They monitor and consider various elevation data, including readings from sensors, the top of the bank, nearby roadway elevations, and upstream and downstream real-time water depth. In extreme events, this assessment allows them to determine whether an emergency decision is warranted.
• The value of this data for the roadway engineers is in establishing flood-level thresholds for the nearby pavements and other relevant infrastructure and using it in conjunction with the sensors' real-time stage data, which forms the basis for an effective flood alert network and informed road closure decision-making.
• This dataset represents the elevation of several critical points near the sensors. So, this data may be valuable for the hydrologists to use with Digital Elevation Models (DEM) and Light Detection and Ranging (LiDAR) to modify the terrain locally right around the location of the sensors (Terrain Modification application in hydrological modeling).
• SRA allowed the project team to configure the data onto a web platform accessible to the public. Flood level thresholds and historical and real-time data are accessible and downloadable for the public and decision-makers on https://setexasrain.onerain.com/ [3]. The platform provides a river-level map and several other functions and features, including access to data from 279 sensors across different agencies.
• The web platform offers a subscription service for alarms, allowing users to receive notifications via email or text after providing their contact details. It also provides additional resources and dashboards. Users can access relevant News and a list of categorized 'Advisories' posts. The platform includes flood monitoring site maps, coordinates, addresses, images, sensor elevation, and water temperature information.
• This data is currently used by the Texas Department of Transportation (TxDOT), Houston Transtar, National Weather Service (NWS), and SRA of Texas. TranStar Flood Warning System is an excellent example of utilizing this data to achieve accurate and reliable results. By combining this data, real-time precipitation data, and traffic information, TranStar identifies flooding risk on roadways and displays the data on TranStar's traffic map and mobile application [4,5].

## 2 Objective

Floods have an enormous impact on society and the economy in the United States, and these effects get worse over time [6]. Harvey caused $125 billion in damage and killed at least 88 people by October 2017 [7,8]. Recent research testified that climate change may result in more flooding and needs a multi-agent decision-making framework [9]. Following Hurricane Imelda, the Southeast Texas Flood Coordination Study (SETxFCS) and the Center for Resiliency (CfR) were established, led by Lamar University, and the group's primary goals have been to increase the resilience of the area and to advance the required knowledge [10,11]. The objective of generating this data is to establish flood-stage thresholds near monitoring locations as part of a real-time flood alert network. This network is used by local, state, and federal agencies to monitor drainage infrastructure and provide the community with flood and roadway closure alerts. The data is also shared with agencies to use within their alert networks. The public can also access real-time updates and knowledge about flood levels during extreme events. Ultimately, this data may lead to faster emergency management response time and evacuation notifications.

## 3 Data Description

The data in files “Southeast Texas Networked Flood Monitoring Sensors.xlsx” (excel file), “Southeast Texas Networked Flood Monitoring Sensors _CSV1.csv”, and “Southeast Texas Networked Flood Monitoring Sensors _CSV2.csv” (comma-separated values text file) contains the measured points from 74 sensor-sites surveyed using Trimble Geo 7X Handheld Device and a ZIPLEVEL PRO-2000 altimeter.

The folder “Southeast Texas Networked Flood Monitoring Sensors _ All Sites _ Shapefile.zip” (zip file), contains multiple necessary ArcGIS physical files, these include the following file extensions: .dbf, .shp, .shx, .cpg, .prj, .sbn, and .sbx. that make up the dataset geographic features. The database table (.dbf) file stores the attribute table. The .shp file is a vector data file format that stores the location, feature attributes, and geometry. The shapefile (.shx) is needed to keep the geometry's index. The .cpg file defines the encoding used to create the shapefile. The projection (.prj) file is used to identify the shapefile coordinates and projection reference. The .sbn and .sbx files help to rapidly and precisely search through spatial features. In this dataset, the titles of the columns in the shapefile attribute table (.dbf) in ArcGIS start with a lowercase letter same as the column headers of the corresponding Excel (.xlsx) and CSV (.csv) files. Furthermore, the folder “Southeast Texas Networked Flood Monitoring Sensors _ Each Site Details _ Shapefiles.zip” (zip file), contains the shapefiles showing each site's measurement details.

The folder “Southeast Texas Networked Flood Monitoring Sensors _ All Raw files _ Surveying” (zip file), contains the field survey raw files directly exported from the Trimble Geo 7X Handheld. This device generates file extensions: .trm, .dd, .gic, .gip, .gis, .giw, .gix, .obs, and .obx while collecting GPS data in the field for each site. These files store the unprocessed GPS data collected by the GPS receiver. The data in these files include the position, time, and other related information, such as satellite constellation information, signal strength, and receiver status [12].

The folder “Southeast Texas Networked Flood Monitoring Sensors _ All Raw files _ .SSF” (zip file), contains field surveying raw files of 67 flood monitoring sites and each location's details. The zip folder includes the .ssf file extensions used by the GPS Pathfinder Office software for storing GPS data. The GPS Pathfinder Office software is used to post-process and manage GPS data collected from different receivers. The .ssf file is created when GPS data is exported from the GPS receiver to the GPS Pathfinder Office software. The file contains information such as the coordinates, elevation, and other parameters collected by the GPS receiver during data collection. The data in the .ssf file can be edited, analyzed, and exported in various formats [12].

Inside the zip folder named “Southeast Texas Networked Flood Monitoring Sensors _ All Raw files _.COR”, there are files with the .cor extension that are used to store differential correction data for raw files collected during field surveying. Differential correction involves comparing GPS data to a more precise source to enhance its accuracy, and the .cor file stores the correction data that can be applied to the GPS data. The GPS Pathfinder Office software offers different correction sources, including base stations or virtual reference stations, to generate differential correction data that can be applied to GPS data gathered in the field. These .cor files can improve the accuracy of GPS data collected in the area [12].

Each of the last four zip folders in the present dataset consists of 67 folders corresponding to the 67 flood monitoring sites’ serial numbers. The other seven sites' information cannot be published due to confidentiality and security considerations.

**Fig. 1:**
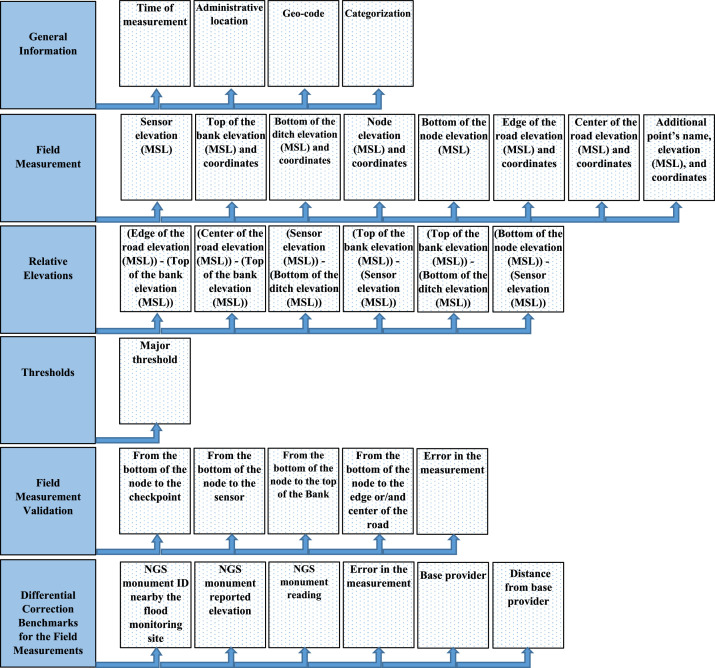
Overview of the dataset organization.

Each water level sensor and node's horizontal positions and vertical elevations were surveyed at each of the 74 sites. Additional surrounding location points were also surveyed, including the top of the bank, the bottom of the ditch, node elevation, elevation of surrounding infrastructure, the edge and center of pavement, and the bottom of the bridge deck. These additional points were surveyed to establish flood-stage alerting thresholds. All variables found within the data files are illustrated in Fig. 1.

(A) General information
  1. Administrative location:
    The name of the agency (owner of the sensor), county name, site ID, sensor address, and the node and sensor manufacturer code.

**Fig. 2:**
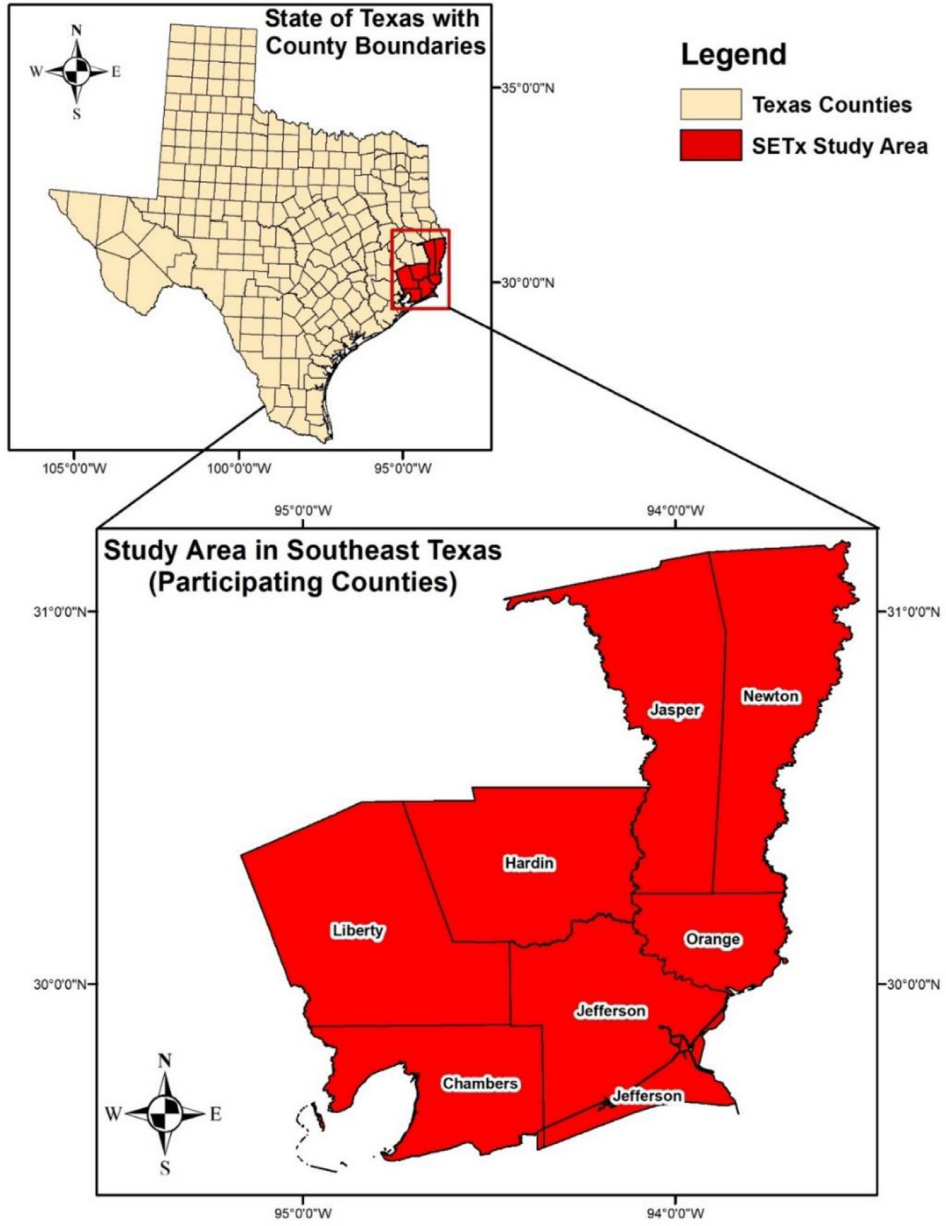
Study area across Southeast Texas.

Fig. 2 shows the location of the study area and seven counties on the southeast edge of Texas, close to Southwest Louisiana, where it covers an area of about 6,000 square miles [13].

**Fig. 3:**
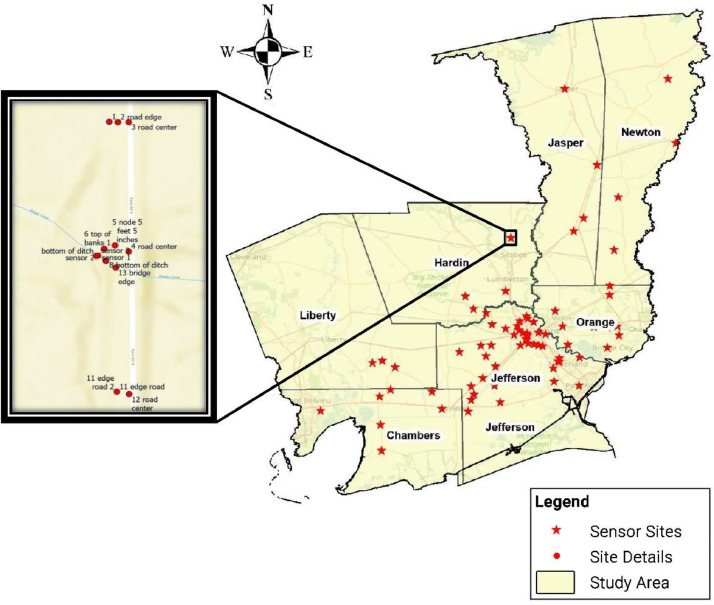
Study area and sensor site locations across Southeast Texas, and the measured points in a flood monitoring site, Hardin County, on ARCGIS PRO 3.

Also, Fig. 3 shows all the flood monitoring sites on an ArcGIS Pro 3 map of Southeast Texas, and the measured points at a flood monitoring site in Hardin County. This dataset includes shapefiles that correspond to the locations of all flood monitoring sites and the detailed measurements of the 67 sites.

2. Time of measurement
  Time and the date of the surveying operations.
3. Categorization
  A waterbody may be called by various local or regional names depending on its location or specific features. The name of each waterbody as the host of the flood sensor was included in this dataset.
  Based on the definition of the watershed delineation, there are three basins in the study area: the Sabine River Basin, Neches River Basin, and Sabine Lake Basin. Each sensor is in one of the mentioned basins.
  Bayous, detention ponds, small earthen ditches, larger earthen ditches, concrete field ditches, drainage ditches, creeks, sloughs, rivers, gullies, long branches, and branches are the main categories of the water bodies related to the project networked sensors.
  For hydrology purposes, specifically, the upstream and downstream specifications are critical when the sensor location needs to be defined as an outlet. Therefore, a brief description of each waterbody as the host of the flood sensor is stated, including the connection of upstream and downstream and the function of the waterbody.
  In this study, each waterbody functions as storage or discharge, which means the sensors' location is subjected to monitoring either the stored or the stage of discharged water after a precipitation event. On the other hand, the relationship between storage and discharge is defined as Rating Curves, which may lead to the next phase of this study to document an economically feasible approach for developing Rating Curves using hydraulic models that would expand the utility of the networked flood sensors in Southeast Texas, allowing the translation of stage readings into discharge estimates.
  In this research area, other bodies of water are often accessible reasonably close or remote to the sensor location. Therefore, their interaction needs to be considered from a hydrological perspective. Accordingly, other elements and features of the categorization in this dataset include the nearest waterbody to the sensor position.
4. Geo-code
  The coordinates (latitude and longitude), datum, and the orthometric height above GEOID18 relative to the NAVD88 vertical datum as the elevation (MSL) of the sensor location. This data is required to identify and locate sensors on maps, GIS-ready maps, and related software accordingly. Furthermore, Top of the Bank latitude, Top of the Bank longitude, Bottom of the Ditch latitude, Bottom of Ditch longitude, Node latitude, Node longitude, Edge of the Road latitude, Edge of the Road longitude, Center of the Road latitude, Center of the Road longitude, Additional Point latitude, and Additional Point longitude are other Geo-codes in the dataset. The additional point is described in the following paragraphs.

(B) Field measurement
1. Sensor elevation (MSL) and coordinates
  The water level sensor (pressure transducer) is positioned near or at the bottom of the ditch and connected (cable within PVC conduit) to a solar-powered transmitting node (transmission via cellular every ten minutes; transmitting node also measures air temperature, barometric pressure, and battery voltage). The water level sensor's horizontal position and vertical elevation were measured at each site. The sensor cable runs parallel to the ditch/waterbody cross-section and is connected to the node.
2. Top of the bank elevation (MSL) and coordinates
  The topmost elevation of the ditch or waterbody containing the water level sensor. The bank's full level, the Top of the Bank, was measured as a point where the body of water is at its highest level or stage when any further rise would cause water to overflow its banks and enter the flood plain. Flooding will be expected if the water level surpasses the top of the bank elevation, and water will spill over onto surrounding areas.
3. Bottom of the ditch elevation (MSL) and coordinates
  The bottommost point of the ditch or waterbody near the water level sensor. The bottom of the ditch, water level sensor, and top of the bank positions are measured along the same cross-sectional plane of the waterbody. The bottom of the ditch, water level sensor, cable, and node run parallel to the cross-section of the waterbody. The bottom of the ditch measurement was recorded to 1) provide relative positioning data between the sensor and the ditch bottom and 2) enable waterbody cross-sectional measurements.
4. Node elevation (MSL) and coordinates
  A solar-powered transmitting device with an IoT (Internet of Things) Module and cellular antenna connected to the water level sensor. Horizontal positions and vertical elevations were recorded to establish the water level threshold at which the flood sensing system would be inundated and cease to function/transmit data. The node is generally positioned 4-10 feet above its foundation.
5. Bottom of the node elevation (MSL)
  The nodes are fastened to a vertical post that is either pile-driven or set into a concrete foundation. A stainless-steel mounting plate is used to attach the node to the post. Also, a waterproof camera is deployed on the post and takes pictures of the site in the requested period. Therefore, when designing thresholds, the node's bottom elevation was recorded for each site.
6. Edge of the road elevation (MSL) and coordinates
  Most sensors are deployed in ditches, streams, and rivers near infrastructure like roadways, bridges, etc. The beginning of the shoulder or curb of the nearby highway is defined as the Edge of the road. The horizontal positions and vertical elevations of these points were recorded. The positions lie along the same plane of the waterbody cross-section containing the bottom of the ditch, water level sensor, and top of the bank positions. The roadway positions and elevations provide information on the severity of the flooding over the roadway, which has implications for any evacuation routing during emergency operations.
7. Center of the road elevation (MSL) and coordinates
  A pavement cross-section is usually center-crowned, in-sloped, or out-sloped. Due to the transverse slope, the centerline of the roadway often has a different elevation from the edge of the road. Therefore, in the case of flooding, the elevation of the center of the road is important in defining the drainage direction. In this project, if a roadway was near the sensor's location, the centerline horizontal positions and vertical elevations were recorded.
8. Additional point's name, elevation (MSL), and coordinates
  Depending on the sensor locations at each site, significant points nearby are necessary for either designing thresholds or confirming the accuracy of measurements. These locations include the infrastructure close to the sensor, such as the bottom of the bridge deck, residential and commercial areas, sidewalks for pedestrians, pipelines, culverts, and schools. In the case of a nearby bridge, if the water level surpasses the elevation of the bottom of the bridge, flooding is expected, and water may inundate the nearby pavement. Also, a nearby precise and well-known position was occasionally chosen and measured as a checkpoint to confirm the accuracy of the obtained vertical elevation using the GNSS surveying. In most sites, the elevation and position of one or more of these points were measured and recorded in the dataset as the Additional Point Elevation, Additional Point Latitude, Additional Point Longitude, and the Additional Point Name.

**Fig. 4:**
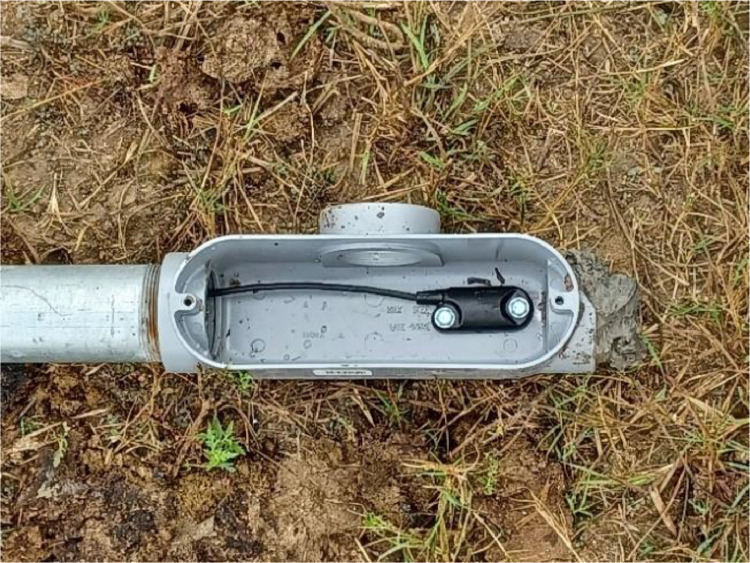
Image of a water level sensor inside of a housing junction positioned at the bottom of a ditch.

An image showing the housing configuration of the water level sensor anchored to the bottom of a ditch is shown in Fig. 4.

**Fig. 5:**
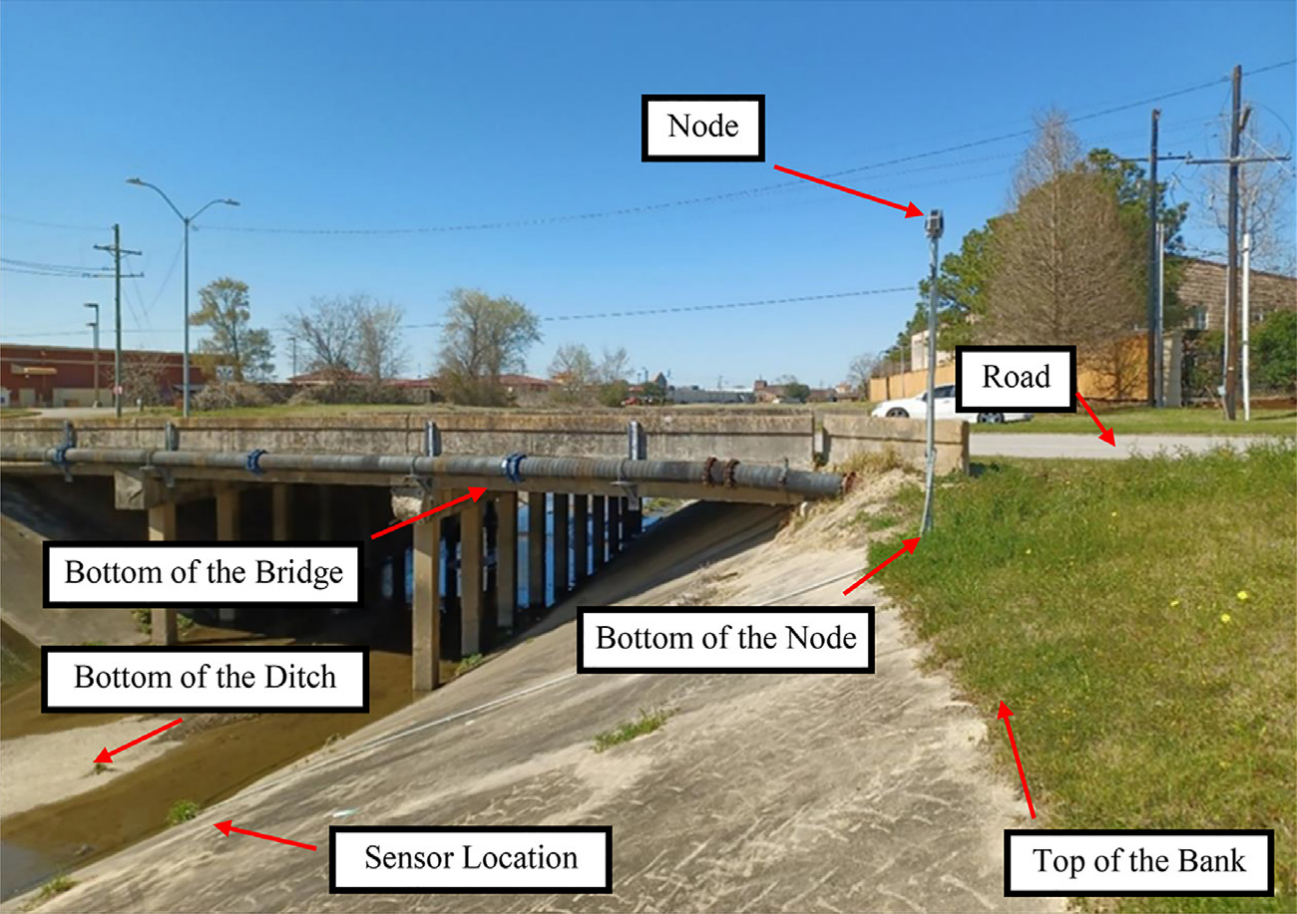
Site Image: Positions where stage thresholds and nearby critical infrastructure are demarcated.

**Fig. 6:**
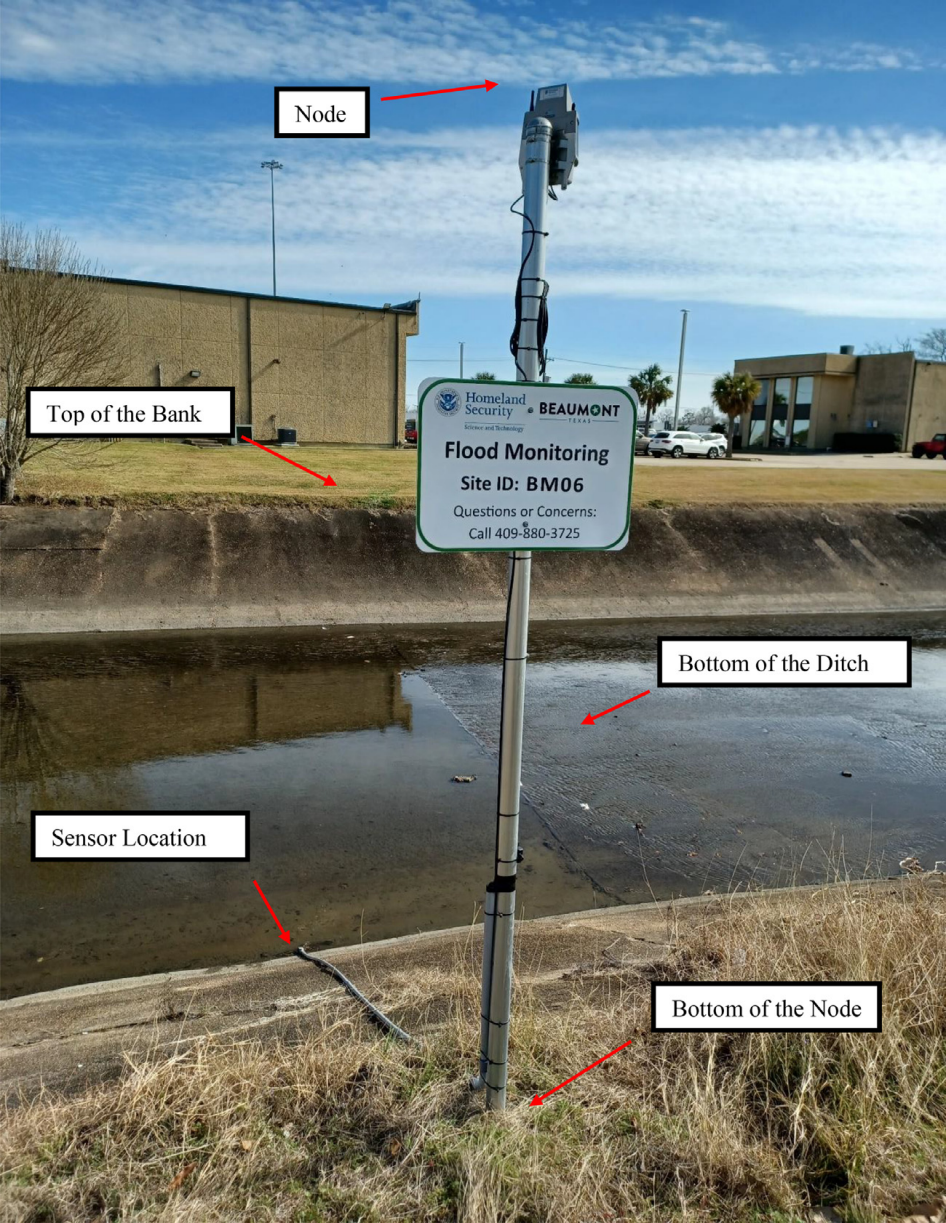
Site Image: Positions where stage thresholds and nearby critical infrastructure are demarcated.

Fig. 5 shows an image of a site and the nearby infrastructure. Also, Fig. 6 is another flood monitoring site in Beaumont, Texas. The positions are demarcated, where survey measurements were taken to establish the sensor elevation and flood stage thresholds.

(C) Relative Elevations
  The dataset also presents the relative elevation data points. This is done for two purposes: i) to confirm the data has been coded correctly into the table (positive results for these relative elevations), and ii) to present the vertical height of the ditch/bank and also present ditch/roadway reference elevations relative to the water level sensor (Sensor Elevation (MSL)) to better assist partnering federal and state agencies.
1. (Edge of the Road Elevation (MSL)) - (Top of the Bank Elevation (MSL))
  In this table column, the elevation of the edge of the available road is subtracted by the elevation of the top of the bank, resulting in a positive number. On the other hand, in a few scenarios, the road is not near the site and may be lower than the top of the bank, hence a negative result for the column.
2. (Center of the Road Elevation (MSL)) - (Top of the Bank Elevation (MSL))
  This column was input with the same method as described in the last paragraph, but the center of the road was used instead of the edge of the road.
3. (Sensor Elevation (MSL)) - (Bottom of the Ditch Elevation (MSL))
  In this system, sensors are installed either on the stream's bed or at a higher elevation along the bank, depending on the site's physical conditions.
4. (Top of the Bank Elevation (MSL)) - (Sensor Elevation (MSL))
  The sensor's function is to measure the water level above it. Sensors are deployed below the bank's top, so this value is positive.
5. (Top of the Bank Elevation (MSL)) - (Bottom of the Ditch Elevation (MSL))
  As a result of the definition of the top of the bank and the bottom of the ditch, these values will be undoubtedly positive.
6. (Bottom of the Node Elevation (MSL)) - (Sensor Elevation (MSL))
  The bottom of the node is the lowest point of the post to which the node was attached. Furthermore, these posts are installed on the top of the banks or close to them. Therefore, the difference between the bottom of the node elevation and the water level sensor is positive.

(D) Thresholds
  Thresholds are the water levels above the sensors described as stages indicating the flood risk at or beyond these elevations. These levels must be exceeded for an agency action, including road closure, alerting, and/or evacuations. The thresholds are also available in the SRA public dashboard online, https://setexasrain.onerain.com/ [3].
1. Major threshold
  The major threshold is the water stage above the sensor, which may result in flooding and emergency measures to address the issue. In flood monitoring sites, a major threshold is defined as the most critical point near the sensor. This study selected important infrastructure including roadways, bridges, and some points in the flood monitoring system, including the top of the bank and bottom of the node as the major thresholds. Furthermore, nearby residential and commercial areas were defined as the major threshold in some places. A description of the major threshold is provided as an attribute in the dataset.

The SRA website allows users to export data within a maximum one-year period, but have an option to access data as far back as 2004. By default, when clicking on a sensor site, the data displayed is for one week. However, users can choose a more extended period, with a maximum of one year, to view historical data. For interested individuals, a user-friendly approach is presented here for accessing real-time and historical data and other valuable information about flood monitoring sites on the public dashboard. A feature ``DHS S&T Sites'' is readily available in the homepage's ``VIEWS'' drop-down menu. Users can access the desired flood monitoring site's complete dataset by choosing this option. The interactive map presents green circles representing available SETxFCS flood monitoring sites, and clicking on them leads the users to the categorized information. These details include metrics such as stage (depth) and flood level thresholds. The stage (depth) page allows users to select specific dates and times or define custom ranges using the drop-down calendar feature. In addition to stage-related data, the public dashboard provides supplementary information, including water temperature, site maps, images and addresses, etc.

**Fig. 7:**
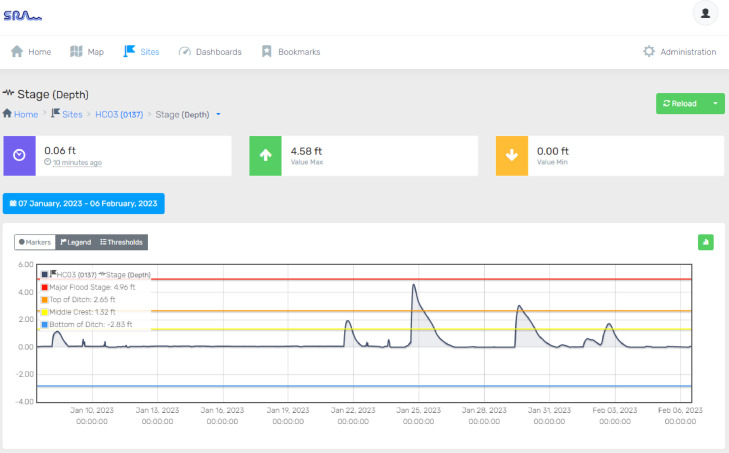
Graphical representation of the water level from 1/10/23-2/6/23 at the sensor site location. The flood stage threshold is superimposed over the water level data. The site is in Hardin County, Texas.

Fig. 7 shows the real-time stages in one month and thresholds for a flood monitoring site in Hardin County on the SRA website, available to the public. As it is shown, interested users may find the real-time water level between the blue line as the bottom of the ditch stage, the yellow line as the additional point stage, the orange line as the top of the bank stage, and finally the red line as the major threshold stage.

**Fig. 8:**
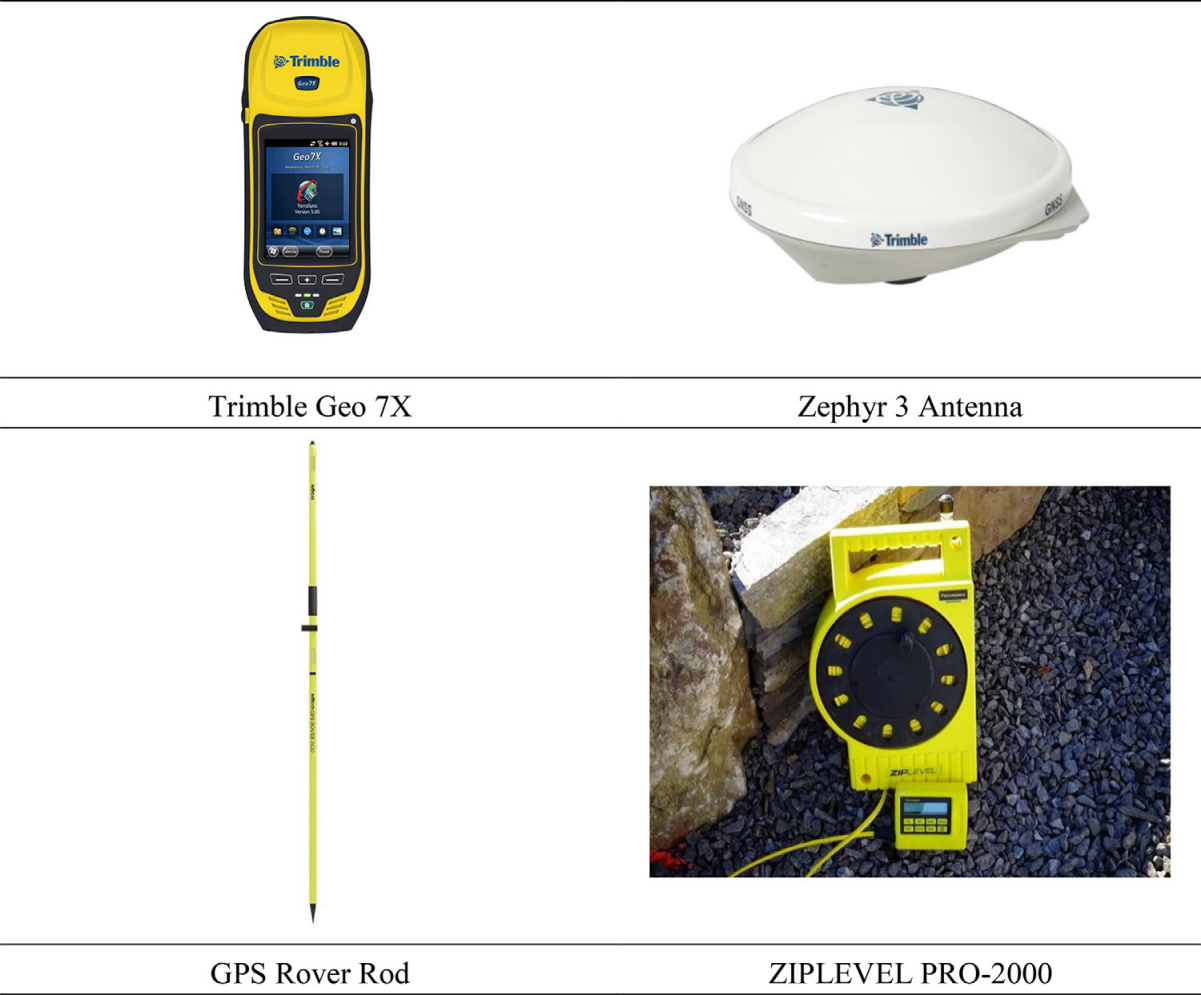
Photos of the surveying tools used in this study.

Also, Fig. 8 shows a compilation of the devices used in this project to achieve the data in field operation [14], [15], [16], [17].

## 4 Experimental Design, Materials and Methods

(A) Study Area

The south-central region of the United States serves as the present research area. The Sabine River and the Neches River are the two most significant of the many rivers and streams that are present in Southeast Texas. It is made up of a marshy area that is very low and flat beside the coast where smaller rivers and creeks congregate to form ``bayous'' that blend in with the surrounding vegetation [18]. Storms serve as a reminder of Harvey and Imelda for the locals who reside in this area. Southeast Texas was chosen as the case study because the Texas Gulf Coast hosts significant transportation corridors through its ports, railways, and highway networks while being vulnerable to catastrophic flooding and heavy rainfall. On the other hand, the area has essential petrochemical, agricultural, and other businesses and thriving educational and cultural resources.

(B) Field Operation

The survey field operation recorded the vertical elevations and horizontal positions of the critical points at the sensor site location, including the sensor, the top of the bank, the bottom of the ditch, the node, and nearby infrastructure-specific points like the edge of the pavement.

The Trimble Geo 7X was turned on to initialize the surveying, and the connection to the satellites was established. The settings were set to the NAD 1983 (2011) as the geographic coordinate system, (NAVD 88) as the vertical datum for orthometric heights established for vertical control, GEOID18 as the latest hybrid geoid model to convert the ellipsoidal height obtained by the Global Navigation Satellite System to the orthometric height of the North American Vertical Datum of 1988 (NAVD 88) within the United States and its territories, and the H-Star Surveying Technology. The Zephyr 3 external antenna was attached to the top of a 2-meter height rover rod. The Trimble Geo 7X settings were adjusted so the elevations and positions were offset and recorded from the bottom of the rod.

Once the Trimble Geo 7X was initialized, in any given instance, there were between 5 and 28 satellites available to connect to the GNSS receiver. Trimble Geo 7X collected GPS and GNSS measurements, satellite positions, and other information into data files over a 60-second interval. The water level sensor elevation was measured using an average of three successive 60-second readings and averaged. At every other location at the site, only one data point was recorded for 60 seconds.

GNSS elevations (at 3-5 different locations at a site) were cross-referenced against elevations obtained using a high-precision hydrostatic altimeter, the ZIPLEVEL PRO-2000, as a near-remote sensing technique to identify measurement errors possibly coming from any obstruction (satellite shadows) and to quantify the scale of GNSS measurement error at valid unobstructed locations. ZIPLEVEL PRO-2000 is a leveling device designed to provide accurate vertical measurements.

The ZIPLEVEL PRO-2000 specifications are summarized below [19,20]:

• Resolution (Internal precision): 0.05”
• Scales: Inches, feet, meters, and centimeters
• Unit dimensions: 16.0” H x 10.5” W x 7.5” D
• Set-up vertical range: 40’ above and below Base Unit
• Set-up horizontal range: 200’ dia. Circle
• Operating temperature: -22°F to +158°F
• Water Resistance: Rain tight; not immersible
• Total weight: 12 lbs.

The ZIPLEVEL PRO-2000 was used in the field instead of a Total Station surveying instrument because it provides us with quick relative elevations for validation purposes. The ZIPLEVEL PRO-2000 operating principles are based on using a Pressurized Reference Chamber (PRC) or Base Unit and a flexible hose (Cord) with a precision sensor (Measurement Module). The PRC maintains constant pressure and is the baseline for all elevation readings. The Cord contains a proprietary liquid and pressured gas attached to the PRC. The Measurement Module detects changes in pressure, which are caused by changes in elevation. The device measures the difference in pressure between the PRC and the point of interest. By measuring the pressure difference, it calculates the relative elevation difference between the reference point and the target location [21,22].

To measure with the ZIPLEVEL PRO-2000, lay the device on the Base Unit on its back. Pull enough hose from the reel to cover the area you need to measure, then turn on the unit. Ensure the device is placed on a known elevation point (a benchmark or a starting reference) and zeroed. This establishes a level reference plane for precise measurements. In this project, the starting point is defined as the bottom of the post to which the node is attached. Then, users can move to different locations of interest and take measurements by placing the Measurement Module on the target surface, featuring negative readings for positions below the reference plane and positive readings for positions above it [21,22].

At the end of each flood monitoring site measurement, a nearby National Geodetic Survey (NGS) benchmark was surveyed and compared against the reported datasheets to further validate the survey positions and elevations.

(C) Field Measurement Validation

In this project, all the obtained vertical elevations in the flood monitoring sites were validated with measurements taken with a ZIPLEVEL PRO-2000 device. This altimeter was used in the field to measure the relative elevations of the points and to compare the results with the GNSS-obtained elevations. The outcome was input into the dataset as the ZIPLEVEL analysis. Checkpoints were defined as the locations of interest where the elevation data was validated with ZIPLEVEL PRO-2000. Checkpoints were selected from various essential locations in the vicinity of the sensors, including the bottom of the bridges, the top of the outfalls, the top of the pipes, and any other critical structures or equipment in the sensor neighborhood.

  1. From the bottom of the node to the checkpoint

This relative elevation is defined as the difference in elevation between the lowest point of the post to which the node is attached and the elevation of the sensor. Moreover, in several sites, the bottom of the node plays the role of the major threshold. Therefore, this relative elevation is critical and needs to be recorded during field operations. The difference in elevation between the bottom of the node and the checkpoint was measured by the ZIPLEVEL PRO-2000; the result is available in the dataset.

  2. From the bottom of the node to the sensor

The difference in elevation between the bottom of the node and the sensor was measured by the ZIPLEVEL PRO-2000, compared to the relevant relative elevation, and was input into the dataset.

  3. From the bottom of the node to the top of the bank

The elevation difference between the bottom of the node and the top of the bank was recorded with the ZIPLEVEL PRO-2000 and compared against the Trimble Geo 7X measurement results. The outcome was included in the dataset.

  4. From the bottom of the node to the edge or/and center of the road

The difference in elevation between the bottom of the node and the edge or/and center of the road was recorded with the ZIPLEVEL PRO-2000 and compared against the relative elevation recorded by the Trimble Geo 7X.

  5. Error in the measurement

The error column is the average of the absolute differences in relative elevations recorded by the ZIPLEVEL PRO-2000 and the relative elevations measured by the Trimble Geo 7X. The error is reported as a percentage.

(D) GPS Pathfinder Office 5.90

The GPS Pathfinder Office 5.90 software was used for data validation, differential correction, and data post-processing.

(E) Differential Correction Benchmarks for the Field Measurements

The GPS Pathfinder Office 5.9 software differentially corrects the field surveying raw (.SSF) files exported from the Trimble Geo 7X device. Several Base Station Providers initiate this data postprocessing operation. These benchmarks are provided in the software online and sorted from the nearest to the furthest to the location of the sensors. Accordingly, choosing the nearest reference for the post-processing operation is highly recommended. Therefore, several benchmarks were examined to achieve the most accurate results. Several National Geodetic Survey (NGS) monuments near the sensor site have been measured and compared to the available NGS published datasheet to further validate the differential corrections process.

  1. NGS monument identification (ID) nearby the flood monitoring site

Several NGS monuments are near each flood monitoring site to validate the surveying operation. In the dataset, the ID of the measured NGS monument is recorded.

  2. NGS monument reported elevation

For each monument, an official NGS report is available on their website [23]. The reported vertical elevation of the selected benchmark is present in the dataset.

  3. NGS monument reading

The GNSS-measured vertical elevation of the monument was input into the dataset. It can be compared to the NGS datasheet to validate the digital surveying operation at each flood monitoring site.

  4. Error in the measurement

After reading the local NGS benchmark, the deviation from the NGS officially published report was expressed in percentage and was input into the dataset.

  5. Base provider

This dataset also reports the name of the selected base providers used in GPS Pathfinder Office 5.9 software for differential correction.

  6. Distance from base provider

For accuracy purposes, in GPS Pathfinder Office 5.9 software, the nearest benchmark to the flood monitoring site must be selected for differential correction. Thus, the distance between the sensor location and the benchmark is reported in the dataset.

(F) ArcGISPro3

All the flood monitoring sites were located on an ArcGIS Pro 3 map in Southeast Texas, and the resulting shapefile and the required physical files for this geographic feature, were provided in the present dataset. Furthermore, the shapefiles relevant to each site's surveying details are available and accessible here to download by interested researchers.

(G) Limitation of the Present Dataset in Hydrological Modeling

In 25 out of the 74 sites, the water in the ditches/rivers was too deep to be measured using the Trimble handheld device. Therefore, an alternative method is needed to measure the bottom of these ditches/rivers. The primary purpose and application of this data set is to develop a flood alert network, rather than for modeling purposes. Modeling is considered a secondary application; not all sites have the necessary information. For many sites, this dataset can be integrated with available DEMs and LiDAR data to enhance the resolution of the terrain and support hydrological modeling.

## CRediT authorship contribution statement

**Hossein Hariri Asli:** Conceptualization, Data curation, Methodology, Software, Validation, Visualization, Writing – original draft, Writing – review & editing. **Nicholas Brake:** Conceptualization, Data curation, Methodology, Project administration, Resources, Supervision, Writing – original draft, Writing – review & editing. **Joseph Kruger:** Conceptualization, Data curation, Methodology, Writing – review & editing. **Liv Haselbach:** Conceptualization, Funding acquisition, Project administration, Resources, Supervision, Writing – review & editing. **Mubarak Adesina:** Data curation, Software, Visualization.

## Data Availability

Southeast Texas Networked Flood Monitoring Sensors (Original data) (Mendeley Data). Southeast Texas Networked Flood Monitoring Sensors (Original data) (Hydroshare).
